# Magnetic Processing of Diamagnetic Materials

**DOI:** 10.3390/polym12071491

**Published:** 2020-07-03

**Authors:** Masafumi Yamato, Tsunehisa Kimura

**Affiliations:** 1 Department of Applied Chemistry, Tokyo Metropolitan University,1-1 Minami-ohsawa, Hachioji, Tokyo 192-0397, Japan; 2 Division of Forestry and Biomaterials, Kyoto University, Sakyo-ku, Kyoto 606-8502, Japan; tkimura@kais.kyoto-u.ac.jp; 3 Fukui University of Technology, 3-6-1 Gakuen, Fukui 910-8505, Japan

**Keywords:** magnetic force, magnetic torque, separation, particle manipulation and patterning, crystallization, magnetic dipole–dipole interaction, orientation, levitation, thermodynamics

## Abstract

Currently, materials scientists and nuclear magnetic resonance spectroscopists have easy access to high magnetic fields of approximately 10 T supplied by superconducting magnets. Neodymium magnets that generate magnetic fields of approximately 1 T are readily available for laboratory use and are widely used in daily life applications, such as mobile phones and electric vehicles. Such common access to magnetic fields—unexpected 30 years ago—has helped researchers discover new magnetic phenomena and use such phenomena to process diamagnetic materials. Although diamagnetism is well known, it is only during the last 30 years that researchers have applied magnetic processing to various classes of diamagnetic materials such as ceramics, biomaterials, and polymers. The magnetic effects that we report herein are largely attributable to the magnetic force, magnetic torque, and magnetic enthalpy that in turn, directly derive from the well-defined magnetic energy. An example of a more complex magnetic effect is orientation of crystalline polymers under an applied magnetic field; researchers do not yet fully understand the crystallization mechanism. Our review largely focuses on polymeric materials. Research topics such as magnetic effect on chiral recognition are interesting yet beyond our scope.

## 1. Introduction

Most polymeric and other materials are diamagnetic. Diamagnetism is well known [1,2,3,4] but was long thought to have little effect on polymeric materials. This perspective began to change 30 years ago, when superconducting magnets became readily accessible to material scientists and engineers. The magnetic field generated by superconducting magnets is 10× stronger than those generated by electromagnets and permanent magnets. The magnetic effect produced by superconducting magnets is 100× larger than that of other magnets because the magnetic energy—the origin of the magnetic effect—is proportional to the square of the intensity of the magnetic field. Interesting applications of superconducting magnets—possible only because of the use of high magnetic fields— include magnetic levitation [5,6,7] and the Moses effect [8]. Researchers have extensively studied the physical [9], chemical [10,11], and biological [12] effects of magnetic fields. The magnetic effects on polymeric materials were applied to polymer processing [13,14].

Most magnetic effects can be understood in terms of the magnetic energy. The magnetic energy of diamagnetic materials is usually very small and hence, may be difficult to disentangle from other energies such as thermal, gravitational, electric, and elastic [15]. In our review, we explain various diamagnetic phenomena in terms of magnetic energy. We cover magnetic effects on diamagnetic materials in general, with a focus on applications to polymeric materials. Research on polymer composites containing ferromagnetic particles [16] is important, but is beyond our scope.

This article is organized as follows: Section 2 presents the magnetic energy origin of the magnetic effect. Although the magnetic energy of diamagnetic materials is much smaller than that of ferromagnetic materials, it is still effective. We discuss the feasibility of the magnetic effect under appropriate conditions. Section 3 provides the basics of magnetic force. We discuss the basic equations for levitation and separation of diamagnetic materials using magnetic force. As an application of magnetic force, we present a two-dimensional pattern formation of fine particles and crystal growth under levitation. Section 4 provides the basics of magnetic torque. We show the basic equation of the magnetic torque, which is the origin of magnetic orientation, and discuss orientation control of materials with uniaxial and biaxial symmetry. We also discuss the time constant of orientation. In Section 5, we focus on magnetic dipole–dipole interactions and review applications of ferromagnetic dipole–dipole interactions. In Section 6, we discuss the effect on phase transition temperature expressed by the Magneto–Clapeyron equation. The temperature change estimated from the equation is extremely small, but researchers observe a larger temperature change in some experiments. Section 7 and Section 8 consist of magnetic orientation under a phase transition—crystallization and microphase separation, respectively. We discuss in detail the origin of the anisotropic structures contributing to orientation. The origin of magnetic orientation under a phase transition is still an open question. Finally, in Section 9, we summarize and present perspectives on magnetic processing of diamagnetic materials.

## 2. Magnetic Energy

The diamagnetic properties of materials are attributable to the magnetic susceptibility, *χ*: approximately −10^−5^ to −10^−6^ for diamagnetic materials and approximately 10^3^ for ferromagnetic materials. A microparticle with volume *V* placed in a magnetic field *B* has a magnetic energy *E*_mag_ expressed by:

(1) $$E_{\mathrm{mag}}=-\frac{\chi VB^{2}}{2\mu_{0}}$$

where *μ*_0_ is the magnetic permeability of vacuum. The magnetic energy of diamagnetic materials is elevated under a magnetic field because *χ* is negative. For example, when *χ* = −10^−6^, *B* = 10 T, and *V* = (0.1 μm)^3^, *E*_mag_ = 4 × −10^−21^ J, which is compared to this energy value to the thermal energy *k*_B_*T* = 4 × −10^−20^ J at *T* = 300 K, where *k*_B_ is the Boltzmann constant. This gives rise to the Boltzmann factor *e*^−10^, indicating that a microparticle of this volume is almost completely repelled against Brownian motion by applying a magnetic field of 10 T [17].

**Figure 1:**
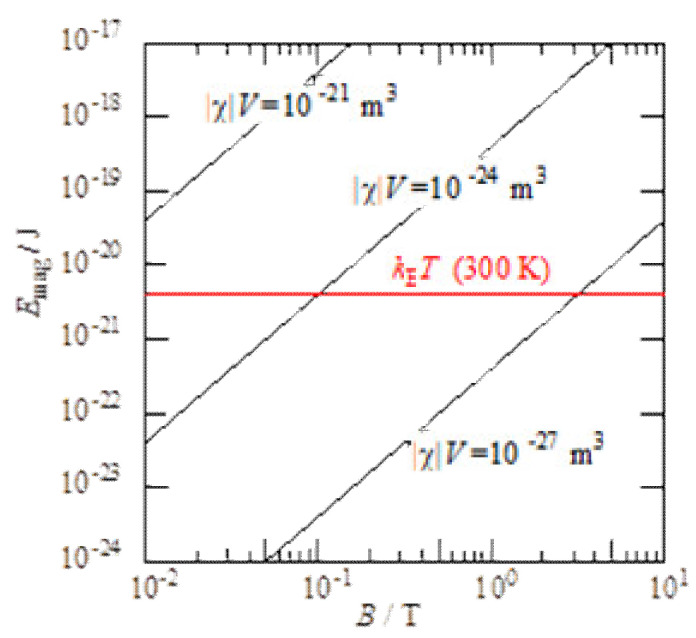
Magnetic energy *E*_mag_ as a function of magnetic field *B*. Thermal energy *k*_B_*T* at 300 K is shown in the figure.

Figure 1 shows the magnetic energy for various values of |*χ*|*V* as a function of the magnetic field [13]. When *B* > 0.1 T, the magnetic energy exceeds the thermal energy if |*χ*|*V* > 10^−24^. Because typical values of *χ* are approximately −10^−6^, *V* > 1 μm^3^. This indicates that microparticles can be manipulated by weak magnetic fields as low as 0.1 T, which electromagnets and permanent magnets readily supply. However, fillers, nucleating agents, and other materials added to polymers are usually larger than the micrometer scale. Thus, we expect that micrometer-scale polymer additives can be manipulated using relatively weak magnetic fields. With much stronger magnetic fields, much smaller additives can be manipulated. Additionally, heterogeneous structures—such as those appearing during crystallization of semi-crystalline polymers and microphase separation of block copolymers—are susceptible to and influenced by the magnetic field. This will be described in detail in the following sections.

## 3. Magnetic Force

If a particle is placed in a spatially inhomogeneous magnetic field, a force is exerted on it. Because the magnetic energy of the diamagnetic particle is elevated in the area where the magnetic field is stronger, the force acts on the particle in a manner that pushes it from a high field area to a low field area. This magnetic force is expressed by:

(2) $$F=-\nabla E_{\mathrm{mag}}=\frac{\chi V}{2\mu_{0}}\nabla B^{2}$$

The intensity of the gradient term is further expressed as (*χ*/*μ*_0_)*BdB*/*dz* in the case of one dimension [18]. The magnetic field gradient generated by a permanent magnet is usually not sufficiently intense to cause a considerable repulsive force—such as that used to levitate diamagnetic particles—because the diamagnetic susceptibility is as small as −10^−6^ [5]. However, a strong magnetic field such as that produced by a superconducting magnet can produce a repulsive force that is sufficient to levitate diamagnetic particles.

### 3.1. Levitation

**Figure 2:**
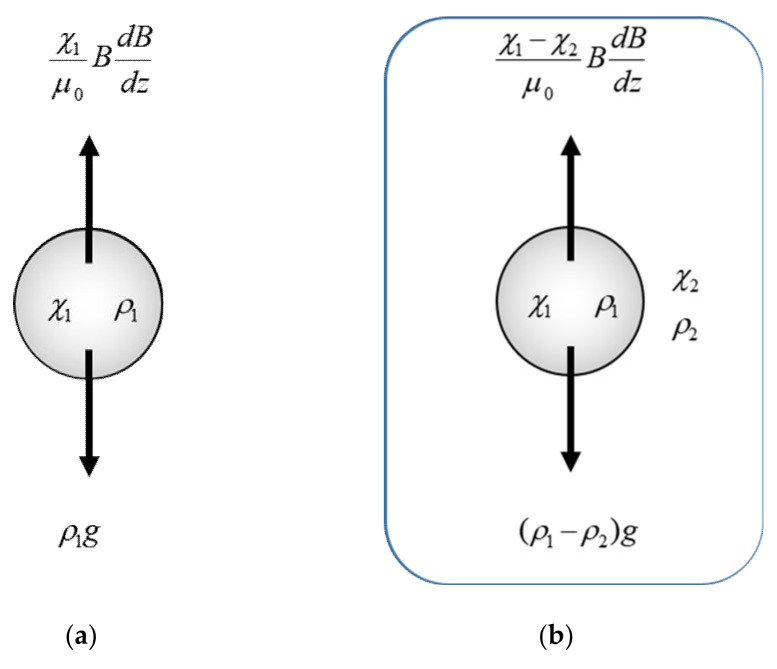
The force acting on a particle with susceptibility *χ*_1_ and density *ρ*_1_ levitating in (**a**) vacuum and in (**b**) a medium with *χ*_2_ and *ρ*_2_.

Consider a diamagnetic particle subjected to a vertical magnetic field gradient. Gravitational and magnetic forces act on the particle. If the magnetic force is sufficiently large to balance the gravitational force, the particle levitates. The corresponding equation (Figure 2a) is expressed by:

(3) $$\rho_{1}g=\frac{\chi_{1}}{\mu_{0}}B\frac{dB}{dz}$$

where *χ*_1_ and *ρ*_1_ are the susceptibility and density of the particle, respectively; *g* is the gravitational acceleration. Some examples are as follows. Researchers have reported magnetic levitation of various organic materials. Water levitates under conditions with a gradient field of *BdB*/*dz* = 1360 T^2^/m that a typical superconducting magnet does not generate. Various areas of science and technology use magnetic levitation [19,20,21,22].

If a particle is surrounded by a liquid medium, two additional forces should be added to Equation (3)—the hydrodynamic and magnetic buoyancies exerted by the liquid medium (Figure 2b). Then, Equation (3) is modified as:

(4) $$\left(\rho_{1}-\rho_{2}\right)g=\frac{\left(\chi_{1}-\chi_{2}\right)}{\mu_{0}}B\frac{dB}{dz}$$

where *ρ*_2_ is the density of the medium (causing the hydrodynamic buoyancy) and *χ*_2_ is the susceptibility of the medium (causing the magnetic buoyancy). The magnetic term in Equation (4) strongly depends on the value of *χ*_2_. Earnshaw’s law shows that *χ*_1_ − *χ*_2_ < 0 should be satisfied for stable levitation. If the medium is paramagnetic or ferromagnetic (*χ*_2_ > 0) and the particle is diamagnetic (*χ*_1_ < 0), this condition is inherently satisfied. Gold is floated in cryogenic oxygen [23]. Researchers regard a paramagnetic particle to be diamagnetic if the particle is immersed in a liquid matrix, where the susceptibility of the matrix is larger than that of the particle. Ikezoe et al. achieved stable levitation of paramagnetic substances (*χ*_1_ > 0) in accordance with Earnshaw’s law [24]. This is termed the magneto Archimedes effect, which is useful when using weak magnetic fields and is widely used to enhance an apparent magnetic force [25,26].

**Figure 3:**
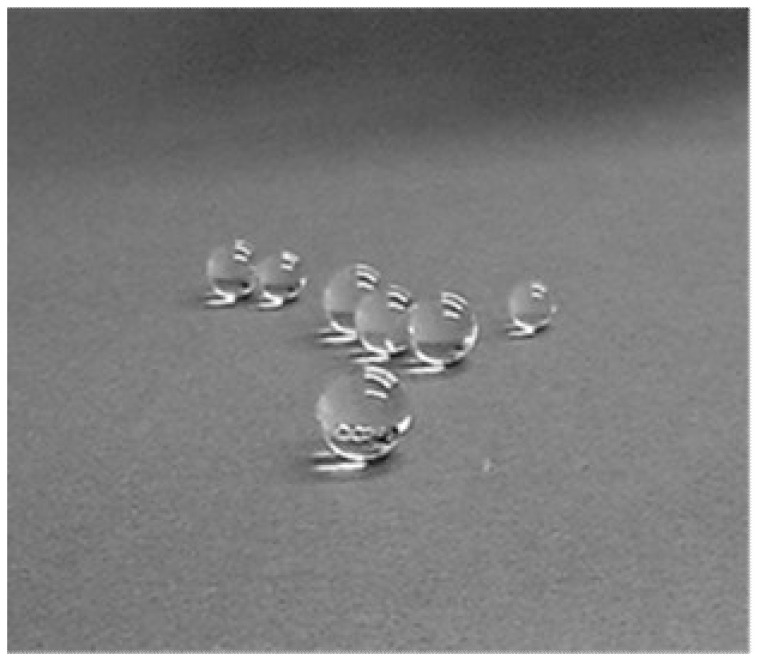
Large poly(benzyl methacrylate) spheres fabricated by levitation polymerization. The size of the sphere is 7 to 9 mm in diameter.

The microgravity environment created by magnetic levitation enables the shape of a liquid droplet to be highly spherical because of non-contacting support. For example, researchers prepared a glass sphere with high sphericity by melting inorganic glass with a laser in a levitation environment [27]. Researchers also polymerized a monomer droplet, levitating in a paramagnetic liquid with a magnetic field of 1 T, to obtain a highly spherical polymer (Figure 3) [28].

### 3.2. Separation

The location of stable levitation of a particle depends on the particle’s density and magnetic susceptibility, and the profile of the magnetic field. Researchers separate polymer pellets—atactic polystyrene, poly(ethylene terephthalate), poly(methyl methacrylate), syndiotactic polypropylene, and styrene–butadiene block copolymer—by using the magneto Archimedes effect in an electromagnet [29]. Researchers have also separated polyethylene and polypropylene [30]. Similarly, researchers have used a permanent magnet to separate polymer particles suspended in paramagnetic solution [31]. Using a superconducting magnet that generates high gradient fields, the separation was possible without paramagnetic media [21]. Attaching magnetic nanoparticles to target diamagnetic particles greatly facilitates separation of the latter [32]. Magnetic levitation enables separation of organic powders, crystal polymorphisms, and chiral/racemic products [33,34,35]. Researchers have non-destructively detected defects in plastic parts [36]. Magnetic levitation is also effective for assembling polymer particles [37].

### 3.3. Particle Manipulation and Patterning

A magnetic force also enables control of mass transfer. The magnetic energy *E*(*x*,*y*,*z*) of a particle suspended in a medium in a magnetic field is expressed by:

(5) $$E\left(x,y,z\right)=-\frac{\chi_{1}-\chi_{2}}{2\mu_{0}}VB{\left(x,y,z\right)}^{2}+\left(\rho_{1}-\rho_{2}\right)gz$$

where *B*(*x*,*y*,*z*) is the magnetic field at the point specified by (*x*,*y*,*z*); the (*ρ*_1_.− *ρ*_2_.)*gz* term is attributable to gravity; *χ* is the magnetic susceptibility; *ρ* is the density; the suffixes 1 and 2 denote the particle and medium, respectively. If one properly combines the parameters in Equation (5) and the distribution of the magnetic field *B*(*x*,*y*,*z*), a minimum of *E*(*x*,*y*,*z*) can be generated at a particular three-dimensional position. Then, particles are bound to this minimum. Several methods have been proposed to generate minima [38]. The spacing of the minima can be as narrow as 100 μm, and micrometer-sized diamagnetic particles can be assembled [39]. By changing the position of the minimum point, researchers can transport the trapped particles.

**Figure 4:**
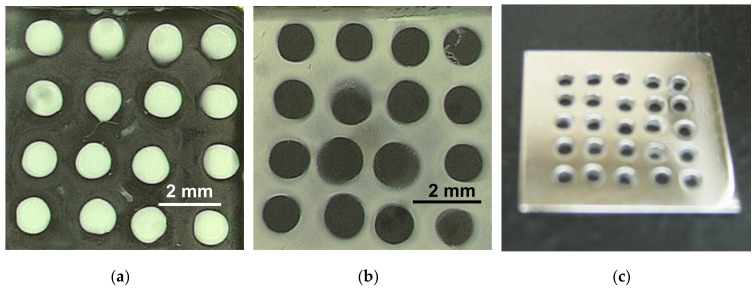
(**a**) Patterned polystyrene nanoparticles (sizes of 1.0 μm, diamagnetic) and (**b**) patterned polystyrene nanoparticles including Eu (sizes of 260 nm, paramagnetic) formed on a glass plate placed on (**c**) permalloy substrate with holes. The direction of the applied magnetic field of 8 T was normal to the substrate.

Figure 4 shows two-dimensional patterns of nanoparticles formed over magnetic minima created by a magnetic modulator consisting of a permalloy that has an array of holes. A glass plate, 130 μm thick, is placed on the magnetic modulator, and the particle dispersion is cast on the plate and dried in a magnetic field of 8 T. Because the magnetic flux preferentially passes through the ferromagnetic substrate, the magnetic flux density over the holes decreases. As a result, the diamagnetic particles collect over the holes and the paramagnetic particles collect elsewhere. Magnetic trapping is promising for contactless manipulation of microparticles and cells [40,41,42].

### 3.4. Crystallization Under Levitation

Researchers often consider magnetic levitation to be an alternative to the microgravity of space, for example, for crystal growth [43,44,45,46]. Molecular crystals are often prepared from solutions. Crystals grow in saturated solutions by molecular deposition onto the surface of pre-formed small crystals. The depletion zone forms by molecular deposition near the crystal surface, where the solution density is lower than in the outer region. Thus, the solution in the depletion zone rises by buoyancy and the depletion zone refreshes with fresh saturated solution flowing toward the surface of the growing crystal. This buoyancy convection has various effects on crystal growth. For example, protein concentration around the crystal is disturbed because of this flow, leading to a deteriorated crystal quality [47]. Similarly, disturbing the concentration distribution of impurities such as protein oligomers around the crystal also deteriorates crystal quality [48]. Furthermore, convection promotes deposits and incorporation of dust particles in the solution onto the crystal surface [49]. However, microgravity suppresses convection, resulting in high quality crystal growth [50]. Researchers have also improved protein crystal quality grown under quasi-microgravity by balancing gravity and magnetic force [43,44,45,46]. In situ observations of crystal growth under magnetic force-based quasi-microgravity provide insight into crystal growth [51].

## 4. Magnetic Torque

### 4.1. Magnetic Anisotropy

The magnetic anisotropy of a molecule arises from the magnetic anisotropy of its chemical bonds. The magnetic susceptibility of a sigma bond is smaller in the direction parallel to the bond axis (*χ*_‖_) than in the direction perpendicular to the bond axis (*χ*_⊥_). In other words, the anisotropic magnetic susceptibility *χ*_a_, defined by *χ*_a_ = *χ*_‖_ − *χ*_⊥_, is negative. Both *χ*_‖_ and *χ*_⊥_ are negative (diamagnetic). Conversely, *χ*_a_, for double bonds such as C=C and C=O, is positive. Aromatic rings have a smaller susceptibility in the direction perpendicular to the ring [52].

Polymeric fibers are elongated in the fiber direction. For example, in polyethylene fibers, the main-chain sigma bonds are elongated in the direction of the fiber axis, and thus, *χ*_a_ < 0 [53]. Here, ‖ and ⊥ indicate the directions parallel and perpendicular to the fiber axis, respectively, whereas *χ*_a_ < 0 for cellulose fibers and nylon fibers, *χ*_a_ > 0 for carbon fibers [53,54,55]. 

In general, the anisotropic magnetic susceptibility of a crystal is expressed by the magnetic susceptibility tensor *χ*. Its principal values are expressed by *χ*_1_, *χ*_2_, and *χ*_3_, and the associated principal axes are termed the *χ*_1_, *χ*_2_, and *χ*_3_ axes. Here, we define *χ*_1_ ≥ *χ*_2_ ≥ *χ*_3_. For isotropic crystals, *χ*_1_ = *χ*_2_ = *χ*_3_. For uniaxial crystals, including trigonal, tetragonal, and hexagonal, *χ*_2_ is equal to either *χ*_1_ or *χ*_3_, where either *χ*_1_ or *χ*_3_ is the major axis. For biaxial crystals, including orthorhombic, monoclinic, and triclinic, the three principal values differ. The magnetic *χ*_1_, *χ*_2_ and *χ*_3_ axes are orthogonal to each other, and they are related to corresponding crystallographic axes [56].

### 4.2. Anisotropic Magnetic Energy

If a particle has magnetic anisotropy, its magnetic energy depends on its orientation relative to the applied magnetic field. This anisotropic magnetic energy causes a torque on the particle. The initial orientation of the particle changes with respect to direction such that the magnetic energy decreases. The particle ends up with the orientation that has a minimum energy. The particle may oscillate before reaching the final orientation depending on the viscosity of the medium suspending the particle. If the viscosity is high enough to overwhelm the inertia term, the rotation of the particle is strongly dampened and oscillation does not occur. 

**Figure 5:**
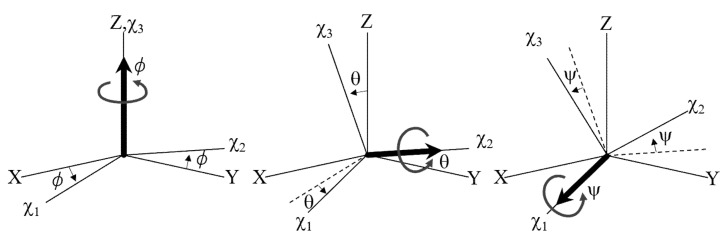
Euler angles *ϕ*, *θ*, and *ψ* that define the relationship between the magnetic *χ*_1_, *χ*_2_, and *χ*_3_ axes and the laboratory coordinates X, Y, and Z. *ϕ*, *θ*, and *ψ* are consecutive rotations about the χ_3_, χ_2_, and χ_1_ axes, respectively. When these angles are infinitesimally small, they correspond to the rotation about the laboratory Z, Y, and X axes, respectively.

The anisotropic magnetic energy of crystal is expressed by [57]:

(6) $$E_{\mathrm{mag}}^{a}={\left(2\mu_{0}\right)}^{-1}B^{2}V\left\{K_{1}\left(\chi_{2}-\chi_{3}\right)\psi^{2}+K_{2}\left(\chi_{1}-\chi_{3}\right)\theta^{2}+K_{3}\left(\chi_{1}-\chi_{2}\right)\varphi^{2}\right\}$$

where *K*_1_, *K*_2_, and *K*_3_ are constants that depend on which type of magnetic field is applied (e.g., static or rotating). In Equation (6), only the quadratic terms of the Euler angles [*ψ*, *θ*, and *ϕ* (Figure 5)] are shown; the isotropic and higher terms are not included. The value of $E_{\mathrm{mag}}^{a}$ reaches a minimum at *ψ* = *θ* = *ϕ* = 0, indicating that the three magnetic axes are aligned. Near this minimum, the torque—for example, about the *z*-axis—is expressed by $-\partial E_{\mathrm{mag}}^{a}/\partial \phi$ .

The magnetic *χ*_1_ = *χ*_2_ = *χ*_3_ axes are closely associated with the crystal axes, and therefore, the alignment of the magnetic axes indicates that the crystal is aligned. To achieve alignment of the magnetic axes, specific magnetic fields must be applied.

### 4.3. Magnetic Orientation of Uniaxial Particles

**Figure 6:**
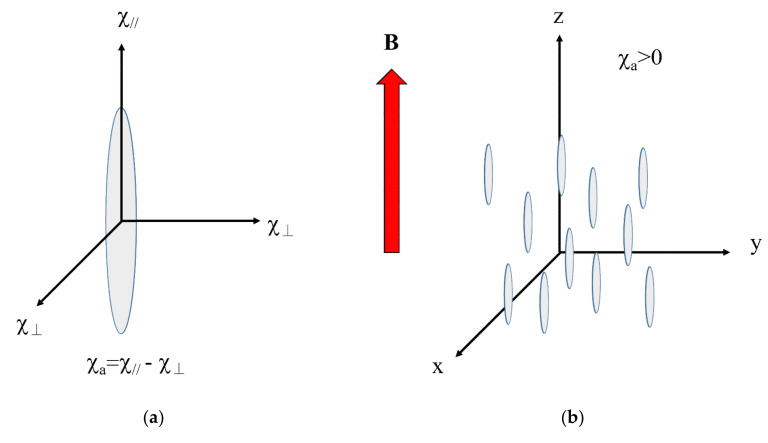
(**a**) Principal axes *χ*_‖_ and *χ*_⊥_ of fiber. (**b**) Schematic drawing of magnetic alignment of fibers with *χ*_a_ > 0 under static magnetic field.

Consider uniaxial particles such as fibers, whiskers, and uniaxial crystals. The magnetic susceptibility of these particles is defined by *χ*_‖_ and *χ*_⊥_, where ‖ and ⊥ indicate the directions parallel and perpendicular to the major magnetic axis, respectively (Figure 6a). The major magnetic axis can be the *χ*_1_ or *χ*_3_ axis. The anisotropic magnetic energy is expressed by:

(7) $$E_{\mathrm{static}}\left(\theta \right)=-\frac{\chi_{a}VB^{2}}{2\mu_{0}}\cos^{2}\theta$$

where *χ*_a_ is the anisotropic magnetic susceptibility defined by *χ*_a_ = *χ*_‖_ − *χ*_⊥_, and *θ* is the angle between *B* and the *χ*_‖_ axis. If *χ*_a_ > 0 (i.e., *χ*_1_ = *χ*_‖_ > *χ*_⊥_), the magnetic energy reaches a minimum when the *χ*_‖_ axis aligns parallel to *B* (*θ* = 0) (Figure 6b). As a result, the *χ*_‖_ axis aligns uniaxially. However, if *χ*_a_ < 0, (i.e., *χ*_3_ = *χ*_‖_ < *χ*_⊥_), the magnetic energy reaches a minimum when the *χ*_‖_ axis aligns perpendicular to *B* (*θ* = *π*/2), the *χ*_‖_ axes assume a planar distribution in the plane perpendicular to *B*.

Fillers are often used in polymer processing. The purpose of adding the filler is to improve physical properties and impart a novel function that is not possessed by the polymer itself. To effectively use the function of the filler, it is important to control the orientation of the filler. However, fillers with shape anisotropy may take undesired orientation during the molding process. Owing to its excellent permeability, a magnetic field is suitable for aligning fillers in a bulk polymer [58,59,60,61,62,63,64]. By magnetic orientation of the filler, we can control the anisotropy of the electrical, optical, mechanical and magnetic properties of the composite materials [58,60,61,63]. For example, vertical alignment of fibrous filler such as carbon nanotubes, which is difficult to achieve by common means, is possible not only in bulk composite material but also in thin film composite material [64]. Table 1 summarizes the magnetic alignment of fillers. Some nucleating agents induce epitaxial growth of polymer crystals due to lattice matching. If such a nucleating agent is aligned by a magnetic field, it is possible to align the crystal of polymers [65]. Uniaxial alignment obtained under magnetic fields is also effective as a means to investigate the epitaxy between nucleating agents and polymers because other effects, such as shear, which orients the molecular chain, are negligibly small under magnetic alignment [66,67].

### 4.4. Magnetic Orientation of Biaxial Crystals

Biaxial alignment of biaxial crystals is possible by using a modulated dynamic magnetic field [17,85]. Staines first proposed this idea, which was subsequently developed by Kimura and Yoshino [86,87]. A simple example is described by:

(8) $$B=\left(B_{x}\cos\omega t,B_{y}\sin\omega t,0\right)$$

where *ω* is the rotational frequency of the magnetic field. If *B_x_* = *B_y_*, the magnetic field is simply a uniform rotation, as described previously. Under the condition *B_x_* > *B_y_* and with a sufficiently large *ω*, the *χ*_1_ and *χ*_3_ axes align in the *x* and *z* directions, respectively. Thus, the three magnetic susceptibility axes are fixed biaxially [87,88]. Because these axes are embedded in a unit cell, the biaxial orientation of these axes indicates biaxial alignment of the crystallographic axes. For uniaxial crystals, where *χ*_2_ = *χ*_1_ or *χ*_3_, only the uniaxial alignment of the major axis is achieved. 

Uniaxial crystals align uniaxially at a maximum regardless of the type of the applied magnetic field because of the uniaxial magnetic nature of these crystals. However, for biaxial crystals (triclinic, monoclinic, or orthorhombic), a much higher alignment, such as biaxial alignment, is possible. The aforementioned amplitude modulation elliptic field (Equation (8)) is one of these choices. Researchers have also proposed methods such as a frequency-modulated elliptic field, intermittently rotating field, and oscillating field [87,88,89,90]. When performing biaxial orientation by means of a modulated magnetic field, the frequency *ω* should be sufficiently high.

Researchers use the biaxial technique for fabricating so called pseudo-single crystals, in turn used to perform single-crystal X-ray and single-crystal solid-state nuclear magnetic resonance analyses from powder samples [91,92]. Researchers also use the biaxial technique to analyze the epitaxial mechanism of polymer crystallization on nucleating agents [93]. Recently, researchers developed an instrument that can generate a linear time-modulated magnetic field for continuously producing biaxial alignment [94].

### 4.5. Alignment Kinetics

**Figure 7:**
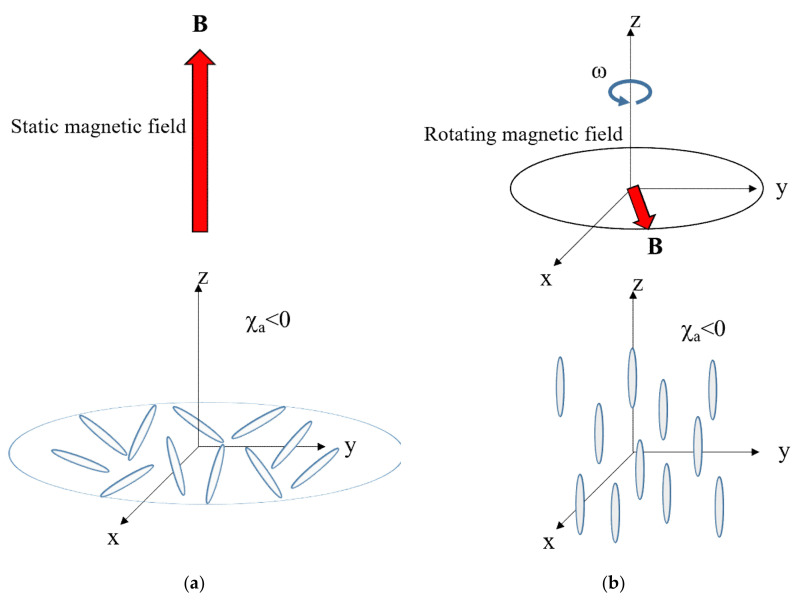
Schematic drawing of applying magnetic fields and expected alignments of the fiber with *χ*_a_ < 0. (**a**) Static magnetic field and (**b**) rotating magnetic field.

If *χ*_‖_‖*χ*_3_, the *χ*_‖_ axes assume a planer distribution in the plane that is perpendicular to a magnetic field (Figure 7a). To achieve uniaxial alignment of the *χ*_‖_ axis, one must use a magnetic field expressed by *B* = *B*(cos *ωt*, sin *ωt*, 0) rotating in the *xy*-plane at a rotation frequency *ω* (Figure 7b). Under this condition, the time-averaged anisotropic magnetic energy is expressed by:

(9) $$E_{\mathrm{rotating}}\left(\theta \right)=-\frac{\chi_{a}VB^{2}}{4\mu_{0}}\cos^{2}\theta$$

where *θ* is the angle between the z-axis and *χ*_‖_ axis. The magnetic energy is half that of the static case because the effective power of *B* is reduced by $\sqrt{2}$ when the magnetic field is rotating.

Consider the orientation kinetics of a uniaxial particle subjected to a static magnetic field. Suppose a particle with a magnetic anisotropy *χ*_a_ is suspended in a medium with viscosity *η* and subjected to a magnetic field *B*. The balance of the magnetic and hydrodynamic torques describes the particle’s rotational motion. Ignoring the inertia term, the equation of motion is expressed by [95,96]:

(10) $$L\frac{d\theta }{dt}=-\frac{\chi_{a}VB^{2}}{2\mu_{0}}\sin2\theta$$

where *θ* is the angle between the magnetic field *B* and the major axis, and *L* is the hydrodynamic resistance. The solution to Equation (10) is given by

(11) $$\tan\theta =\tan\theta_{0}\exp\left(-t/\tau \right)$$

where the alignment rate *τ*^−1^ is defined by *τ*^−1^ = |*χ*_a_|*VB*^2^/(*Lμ*_0_) [53]. For of a sphere with radius *a*, L = 8*πηa*^3^ and *V* = 4 *πa*^3^/3; thus,

(12) $$\tau^{-1}=\frac{\left|\chi_{a}\right|B^{2}}{6\eta \mu_{0}}$$

**Figure 8:**
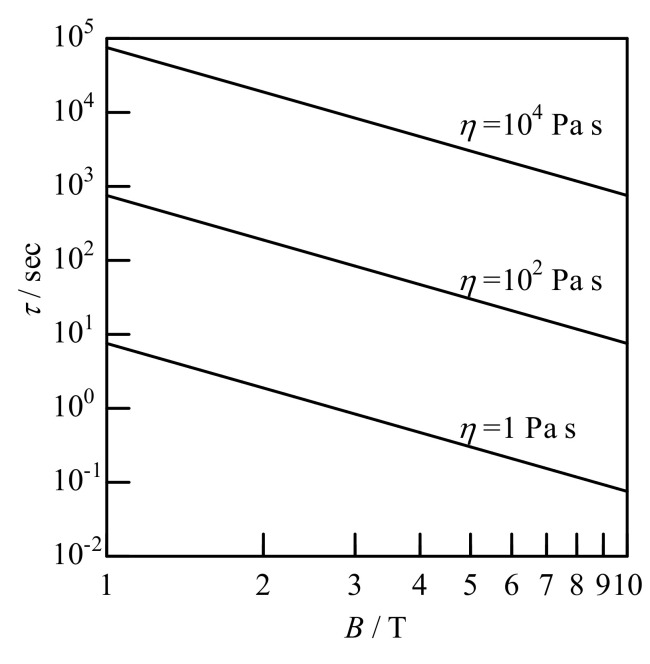
Alignment rate *τ* as a function of magnetic flux density *B* under anisotropic diamagnetic susceptibility of |*χ*_a_| = 10^−6^. Viscosity of media is shown in the figure.

Regarding Equation (12), the alignment rate does not depend on the volume of the particle. This is valid when the particle size is sufficiently large such that Brownian motion can be ignored [17,97]. In a rotating magnetic field, *τ*^−1^ is halved because the effective magnetic field is reduced by $\sqrt{2}$ as a consequence of rotation. Figure 8 shows the relationship between *τ*^−1^ and *B* estimated using Equation (12), where we assume the anisotropic diamagnetic susceptibility is *χ*_a_ = 10^−6^. Magnetic alignment is very fast in a low-viscosity environment such as water.

## 5. Magnetic Dipole–Dipole Interaction

Materials in a magnetic field are magnetized and form magnetic dipoles. The dipole–dipole interaction *E*_12_ between two magnetic dipoles, *μ*_1_ and *μ*_2_, is expressed by Equation (13).

(13) $$E_{12}=-\left(\frac{\mu_{0}}{4\pi }\right)\left(\frac{3\left(\mu_{1}\cdot r\right)\left(\mu_{2}\cdot r\right)}{r^{5}}-\frac{\left(\mu_{1}\cdot \mu_{2}\right)}{r^{3}}\right)$$

where *r* is a distance between the dipoles.

**Figure 9:**
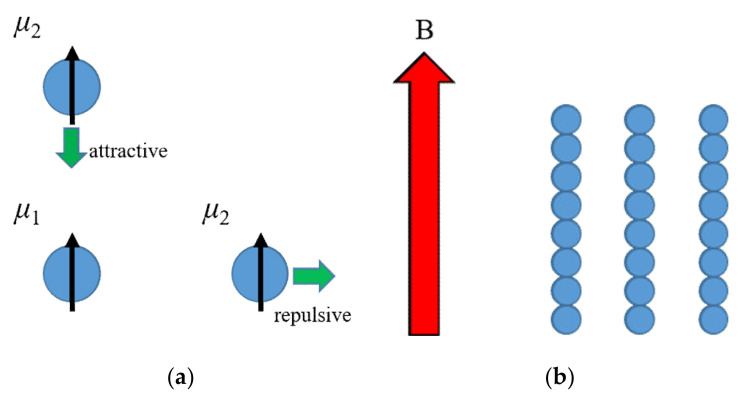
Dipole–dipole interaction acts on the adjacent magnetic dipoles. (**a**) Depending on the arrangement between the dipoles, the direction of the force is different. (**b**) The column structure is formed under a magnetic field.

The force acting between the dipoles may be attractive or repulsive, depending on their mutual location and orientation (Figure 9), resulting in aggregates. The force that is attributable to the diamagnetic dipole moments is extremely small and researchers report only a few examples [98,99,100,101].

The dipole interaction between ferromagnetic dipoles, however, is very intense. Ferromagnetic particles dispersed in a polymer matrix can be assembled and aligned into a column and/or chain structure by an external magnetic field. The alignment brings about anisotropy in conductivity, magnetic susceptibility, thermal conductivity, permittivity, magnetoresistance, and piezoresistivity [102,103,104,105,106,107,108,109]. Particles form aligned chains from a dilute isotropic system by coagulation, which leads to a conductivity jump and increased transparency in the alignment direction [110,111]. The column structure influences the chain orientation of the matrix polymer, resulting in a change in the physical properties of the composite material [112]. The column structure and the structure change in the matrix polymer both affect the physical properties of the composite materials. Researchers also use dipole–dipole interactions to control physical properties by applying magnetic fields. A typical example is magnetic fluid that is a liquid in which ferromagnetic nanoparticles are highly dispersed [113,114,115]. Applying a magnetic field enhances the interactions between the particles in the fluid, thereby increasing the viscosity. The magnetic fluid retained in a gap by a magnetic force acts as a liquid O-ring. Thus, magnetic fluids can be used as a seal [116].

Researchers reported a magnetic composite material, in which magnetic fine particles are dispersed in a hydrogel [16]. This is an example of a stimuli-responsive functional material. The elastic modulus of this material increases by 500× in response to the magnetic field.

## 6. Thermodynamics: Phase Transition Temperature

In ferromagnetic materials, one can modulate the phase transition temperature by applying a magnetic field [117]. However, it is difficult to observe this effect for diamagnetic materials because the magnetic energy received by diamagnetic materials is extremely small. There are several studies regarding thermal analysis of organic compounds in a strong magnetic field [118,119]. The transition temperatures shifted to higher temperatures by several tens of millikelvin by applying a field of 5 T. 

**Figure 10:**
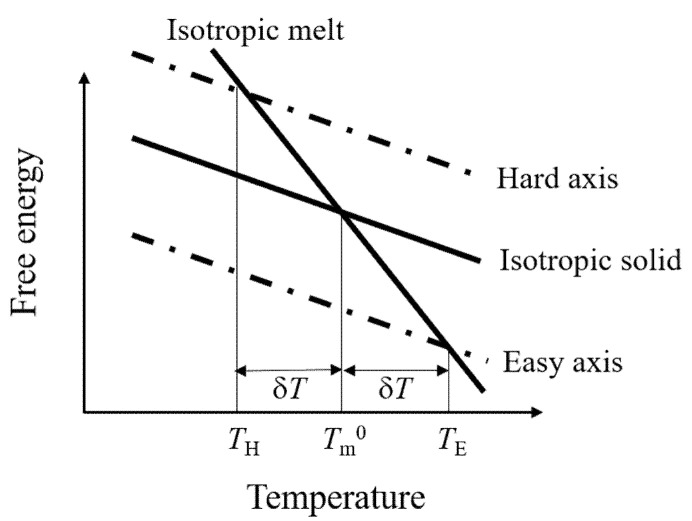
Temperature dependence of free energy. In anisotropic solids, free energy in a magnetic field differs depending on the orientation direction, leading to the orientation dependent shifts of melting point.

The Magneto–Clapeyron equation expresses the value of the temperature shift that is attributable to the magnetic field [120]:

(14) $$\Delta T=\frac{\left\{\left(\cos^{2}\theta -1/3\right)\chi_{a}+\langle \chi_{s}\rangle -\chi_{l}\right\}B^{2}}{2\mu_{0}\Delta \bar{H}}T_{m},$$

where $\Delta \bar{H}$ is the molar transition enthalpy; *χ*_a_ = *χ*_‖_ − *χ*_⊥_ is as defined previously, $\langle \chi_{s}\rangle =(2\chi_{⊥}+\chi_{∥})/3$; *χ*_1_ is the magnetic susceptibility of liquid; *T*_m_ is the melting temperature in the absence of the magnetic field; $\langle \chi_{s}\rangle$ is the average of the phase transition temperature in the absence of the magnetic field; *θ* is the angle between the applied magnetic field and easy axis (here, we assume *χ*_‖_ is easy axis). In accordance with Equation (14), the melting point shifts to a higher temperature when the easy axis of magnetization is aligned parallel to the magnetic field (Figure 10). However, the estimated ∆*T* is on the order of millikelvins, even if $\Delta \bar{H}$ for the isotropic–anisotropic transition for the liquid-crystal is assumed [120]. ∆*T* is much less if $\Delta \bar{H}$ for the melting crystals is assumed. For crystals and liquid-crystal systems $\Delta \bar{H}$, is sufficiently large that it is difficult to observe considerable values of ∆*T*. Researchers studied the magnetic field effect on the order–disorder transition of a diblock copolymer using in situ X-ray scattering, but did not detect a considerable ∆*T* [121]. However, researchers reported a larger ∆*T* for another order–disorder transition [122]. Recently, researchers reported that the crystallization temperature of polyethylene and polyethylene glycol increases by several degrees in a magnetic field [123]. Likely, a high ∆*T* strongly depends on the $\Delta \bar{H}$ of the system under investigation.

## 7. Magnetic Orientation of Crystalline Polymers

Crystalline polymers undergo orientation during crystallization from the molten state in the presence of high magnetic fields. Table 2 shows polymers that researchers have to date reported to align [124,125,126,127,128,129,130,131,132,133]. Atactic polymers do not undergo magnetic orientation because they lack an ability to form anisotropic ordered structures. Incidentally, fibers consisting of atactic polymers can align because the elongated chains exhibit magnetic anisotropy in the direction of the fiber axis. Both aromatic and aliphatic polymers undergo magnetic orientation, indicating that the intensity of the magnetic anisotropy of the constituent monomers is less important. Three factors determine the alignment—the magnetic anisotropy of the monomer, the secondary structures of the polymer chain, and packing in the crystal. All of these factors contribute to the magnetic anisotropy of the crystal.

**Figure 11:**
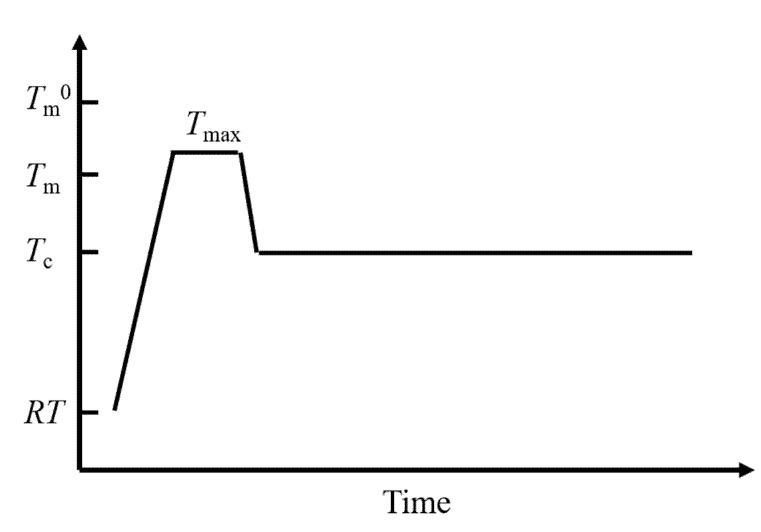
Typical thermal history necessary for the magnetic alignment of crystalline polymers. *T*_m_^0^—equivalent melting point; *T*_m_—melting point; *T*_max_—melting temperature; *T*_c_—crystallization temperature.

Figure 11 shows a typical thermal history used to obtain magnetic orientation. We maintained a polymer sample in a magnetic field, at a temperature between the melting temperature (*T*_m_) determined by differential scanning calorimetry and the equilibrium melting temperature (*T*_m_^0^). Then, we subjected the sample to isothermal crystallization in the magnetic field at a temperature below *T*_m_. The polymer chain orientation can easily occur by shearing strain, temperature gradient, etc. Therefore, these factors should be carefully removed so that the magnetic effect on orientation can clearly observed.

The mechanism of the magnetic orientation of polymeric materials is complicated compared with that of solid particles suspended in a liquid medium, because the magnetic process is coupled with the crystallization process. Furthermore, polymer crystallization itself remains contentious; researchers have proposed various crystallization mechanisms [134].

Two models for the orientation mechanism are as follows. The rotation model is a simple analogue to the magnetic orientation of solid particles in suspension. This model assumes that some ordered anisotropic structures rotate because of the magnetic torque. Here, the ordered anisotropic structures are not limited to crystals but may be, for example, mesophases, liquid-crystalline phases, conformationally disordered phases, crystal embryos, or microcrystals, formed at an early stage of crystallization when the viscosity is not yet substantially high.

In the preference-growth model, the ordered anisotropic structure that happens to have *χ*_1_-axis (easy axis) alignment has a melting temperature that is higher by ∆*T* compared with the isotropic structure, in accordance with the aforementioned Magneto–Clapeyron equation. Thus, under supercooling, the *χ*_1_-axis-aligned structure has higher supercooling compared with the isotropic structure, resulting in preferential structure formation. As discussed previously, ∆*T* might be very small for the crystalline phase, yet large for mesophases because of its small ∆*H*.

In situ birefringence measurement of a polymer melt undergoing crystallization shows birefringence that can be seen at an early stage of crystallization, where the crystals detectable by X-ray diffraction were not yet formed [129]. The origin of the birefringence is attributable to the oriented anisotropic structures that are not the crystal.

Researchers have investigated the origin of anisotropic structures [135]. The orientation degree and crystal texture largely depended on the temperature *T*_max_ (Figure 11) to which the specimen was exposed before being subjected to isothermal crystallization. Researchers achieved higher alignments when the specimen was treated at lower *T*_max_ values. Upon treatment at a high *T*_max_, researchers obtained only a small number of large spherulites, without orientation. Similar observations have been reported elsewhere [130]. Magnetic orientation competes with spherulite formation. However, upon treatment at a low *T*_max_, the sample exhibited a large number of small and oriented crystallites.

These observations suggest that the origin of the anisotropic structure is related to residual microcrystals that remain even above *T*_m_. Usually, researchers heat the sample from room temperature, and the sample undergoes cold crystallization. As a result, the sample contains a number of microcrystals before it reaches *T*_m_. Of course, the remaining microcrystals assumed here cannot be in a form of crystal detectable by X-ray diffraction, but they may be in a form of partially molten disordered crystals, which may be called, for example, mesophase or liquid crystalline phase. Researchers do not yet know whether these structures are stable in equilibrium or only kinetically. If the heat treatment temperature *T*_max_ is higher than the equilibrium melting temperature (*T*_m_^0^), the residual structure no longer remains and there is no magnetic orientation. Commercially available crystalline polymers including poly(ethylene terephthalate), poly(ethylene), nylon, and poly(propylene) undergo magnetic orientation during crystallization from melts (Table 2).

## 8. Magnetic Orientation of Microphase-Separated Structure

Researchers use nanoscale structures formed by microphase separation of block copolymers as templates for developing functional materials [137]. Magnetic alignment also occurs in microphase separation of block copolymers. Researchers can use magnetic alignment to obtain a uniform alignment over a large area.

As mentioned previously, crystalline polymers undergo magnetic alignments. The crystalline and liquid-crystalline blocks of block copolymers exhibit magnetic orientation [138,139]. Researchers have reported magnetic orientation of nanoscale structures with cylindrical and lamellar morphology [140,141,142,143,144,145,146,147,148]. The liquid-crystalline block does not necessarily ensure magnetic orientation of nanostructures [142]. Even if the block copolymer contains an anisotropic component such as a liquid-crystalline and crystalline block, a magnetic anisotropy energy that is greater than the thermal energy and a low viscosity environment that is rotatable by magnetic torque are necessary for magnetic orientation of nanoscale structures.

Osuji and colleagues successfully magnetically aligned a microphase-separated structure formed by a block copolymer consisting of amorphous polymers [149]. Generally, the chains of amorphous polymers form a random coil, resulting in an isotropic structure. It is not immediately obvious why anisotropic polymers can undergo magnetic alignment. An explanation may be molecular orientation at the interface of the microphase-separated structure, or Magneto–Clapeyron type effect occurring with small transition enthalpy.

## 9. Conclusions

We reviewed magnetic phenomena of diamagnetic materials, focusing mainly on polymeric materials. Phenomena such as magnetic levitation, separation, and manipulation of particles originate from the magnetic force, whereas phenomena such as orientation (e.g., crystals and fibers) originate from the magnetic torque. Both the force and torque derive from well-defined magnetic energy. Hence, the phenomena caused by these factors are clearly understood. For example, the magnetic orientation of fillers in polymeric materials is clearly attributable to the action of the magnetic torque on the filler.

The magnetic energy comes into play as an enthalpy term in the free energy when materials are undergoing a phase transition. Crystalline polymers undergo magnetic orientation during crystallization from melts. Additionally, microstructures of block copolymers align under a magnetic field. The alignment mechanism in these systems may be described by preference-growth or rotation of the ordered structure. It is sometimes difficult to determine which factor is dominant because of researchers’ imperfect knowledge of the phase transition of these systems.

Strong magnetic fields are surely optimal compared with weak magnetic fields for maximizing the magnetic effect. Recently, strong magnetic fields such as 10 T or more have become readily accessible to materials scientists and engineers. Although they are not very strong, neodymium magnets of approximately 1 T are much more readily accessible and useful for magnetic processing in many cases. A magnetic field of these strengths is as powerful as an electric field for particle alignment, and superior to electric fields in terms of penetrability.

In addition to the intensity, special and temporal modulations of magnetic field are promising for expanding the potential of magnetic fields for processing. With special modulation ranging from macroscale to microscopic orders, various force fields can be created. With temporal modulation, precise three-dimensional alignment of microcrystals is possible, which will be useful for materials science as well as X-ray diffraction, neutron diffraction, and solid-state nuclear magnetic resonance analyses of microcrystalline powders.

The future prospects of magnetic applications can be summarized as follows:

Intensity—Compared with weaker magnetic field strengths, stronger magnetic field strengths are better. However, users must wait for the development of superconducting technology. The stronger the magnetic field, the smaller the available space. Currently, 10T/100 mm*ϕ* and 5T/400 mm*ϕ* are commercially available. A pulsed magnetic field is one option when high intensity is required and duration can be sacrificed somewhat. It is necessary to devise an approach to utilize the magnetic field under such restrictions.

A strong magnetic field is advantageous for orientation, especially when the oriented samples are to be subjected to single-crystal analyses by X-ray diffraction, neutron diffraction, and nuclear magnetic resonance. However, for micrometer-scale particles, weaker magnetic fields such as those supplied by a neodymium magnet (~1T) are sufficient for alignment, indicating widespread applications in materials science.

Spatial modulation—Regarding separation, the magnetic force (~∆*χBdB*/*dz*) plays an important role. In such circumstances, a weak *B* is sufficient to create a large magnetic force if a large magnetic field gradient *dB/dz* is produced. Because of the divergent nature of magnetic fields, a weak *dB/dz* is ubiquitous. A spatially modulated magnetic field, produced over a microscopically designed bumpy surface of a ferromagnetic material placed in a magnetic field, might be useful for microscopically patterning microparticles and nanoparticles. If one immerses diamagnetic particles in a ferromagnetic or paramagnetic fluid, the difference ∆*χ* between the particle and medium is substantial, resulting in a large magnetic force. Micropatterning can be applied to a wide range of microparticles, ranging from ceramics to cells. A limitation of this technique is that the field modulation persists only over a very short distance—proportional to the pitch of the bumps on the ferromagnetic surface.

Temporal modulation—A time-dependent magnetic field (TDMF) is a promising means to expand the utility of magnetic fields. Three-dimensional alignment of microcrystals is an example. More fundamentally, from the Maxwell equation, rotE=−∂B/∂t, the TDMF essentially induces the electric field *E*. Therefore, the phenomena observed under TDMF are a result of simultaneous application of magnetic and electric fields. The magnitude of *E* is proportional to the frequency *ω* of *B*; thus, *E* may be large if *ω* is as high as the frequency of light. The potential of TDMF seems unexplored currently.

Effect on phase transition—In accordance with the Magneto–Clapeyron equation, the shift of the transition temperature ∆*T* is inversely proportional to the transition enthalpy ∆*H*. This indicates that a very weak first-order phase transition may be very susceptible to the applied magnetic field. Phase separation of block copolymers, phase changes of surfactant solutions, and structure formation during the incubation period of crystallization may fall into the category of weak first-order phase transitions. At present, few studies have focused on these aspects of the magnetic effect.

## Figures and Tables

**Table 1:** List of magnetically aligned fillers. Orientation direction shows the relationship with a static magnetic field.

Filler (Shape)	Orientation Direction
Carbon Fiber/ Nanotube (fiber) [59,68,69,70]	Fiber axis ‖ *B*
Cellulose (fiber) [54,55]	Fiber axis ⊥ *B*
Graphite/ Graphene (sheet) [71]	Plane-normal ⊥ *B*
Graphene oxide (sheet) [72,73]	Plane-normal ⊥ *B*
Hexagonal Boron Nitride (sheet) [74]	Plane-normal ‖ *B*
Aluminum Nitride (sheet) [75]	Plane-normal ⊥ *B*
Mica (sheet) [76]	Plane-normal ⊥ *B*
Montmorillonite(sheet) [77,78]	Plane-normal ⊥ *B*
Talc (sheet) [79]	Plane-normal ⊥ *B*
Nontronite (sheet) [80]	Plane-normal ⊥ *B*
Niobate(V) nanosheet (sheet)[81]	Plane-normal ⊥ *B*
Titanate(IV) nanosheet (sheet)[81]	Plane-normal ‖ *B*
Hydroxyapatite (fiber)[82]	Fiber axis ⊥ *B*
Mordenite zeolite (powder) [83]	Orthorhombic *b*-axis ‖ *B*
PZT (powder) [84]	Rhombohedral *c*-axis ⊥ *B*

**Table 2:** List of magnetically aligned crystalline polymers during melt crystallization. Orientation direction shows the relationship with a static magnetic field.

Polymer (Crystal System)	Orientation Direction
Polyethene-2,6-naphthalate (triclinic) [136]	*c*-axis ‖ *B* (approximately)
Bisphenol A Polycarbonate (orthorhombic) [125]	*c*-axis ‖ *B*
Polyethylene terephthalate (triclinic) [126]	*c*-axis ⊥*B* (approximately)
Polyethylene (orthorhombic) [127]	*c*-axis ⊥*B*
Isotactic Polypropylene (monoclinic) [128]	*c*-axis ⊥*B*
Isotactic Polystyrene (hexagonal) [129,130]	*c*-axis ⊥*B*
Syndiotactic Polystyrene (orthorhombic) [131]	*c*-axis ⊥*B*
Nylon 6 (monoclinic) [132]	*c*-axis ⊥*B*
Poly-l-lactide (orthorhombic) [133]	*c*-axis ⊥*B*
